# Enhancing healthcare IoT systems for diabetic patient monitoring: Integration of Harris Hawks and grasshopper optimization algorithms

**DOI:** 10.1371/journal.pone.0301521

**Published:** 2024-05-29

**Authors:** Mehdi Hosseinzadeh, Zohre Arabi, Saqib Ali, Hong Min, Mazhar Hussain Malik

**Affiliations:** 1 Institute of Research and Development, Duy Tan University, Da Nang, Vietnam; 2 Department of Computer Science, University of Human Development, Sulaymaniyah, Iraq; 3 Computer Engineering and Information Technology Department, Payam Noor University, Tehran, Iran; 4 Department of Information Systems, College of Economics and Political Science, Sultan Qaboos University, Al Khoudh, Muscat, Oman; 5 School of Computing, Gachon University, Seongnam, Republic of Korea; 6 School of Computer Science and Creative Technologies College of Arts, Technology and Environment (CATE) University of the West of England Frenchay Campus, Stoke Gifford, United Kingdom; Nottingham Trent University School of Science and Technology, UNITED KINGDOM

## Abstract

The integration of the Internet of Things (IoT) in healthcare, especially for people with diabetes, allows for constant health monitoring. This means that doctors can watch over patients’ health more closely, making sure they catch any issues early on. With this technology, healthcare workers can be more accurate and effective when keeping an eye on how patients are doing. This not only helps in keeping track of patients’ health in real-time but also makes the whole process more reliable and efficient.By implementing appropriate routing techniques, the transmission of diabetic patients’ data to medical centers will facilitate real-time and timely responses from healthcare professionals. The grasshopper optimization algorithm is employed in the proposed approach to cluster network nodes, resulting in the formation of a network tree that facilitates the establishment of connections between the cluster head and the base station. After identifying the cluster head and establishing the clusters, the second stage of routing is implemented by employing the Harris Hawks optimization algorithm. This algorithm ensures that the data pertaining to diabetic patients is transmitted to the treatment centers and hospitals with minimal delay. For node routing, the optimal next step is selected based on the parameters such as the residual energy of the node, the ratio of delivered data packages, and the number of the neighbors of the node. To continue, first, the MATLAB software is utilized to simulate the proposed method, and then, it is compared with other similar methods. This comparison is conducted based on various parameters, including delay, energy consumption, network throughput, and network lifespan. Compared to other methods, the proposed method demonstrates a significant 33% improvement in the average point-to-point delay parameter in the subsequent iterations or rounds.

## 1. Introduction

The IoT represents a type of wireless network where everything, including people, animals, and even non-living items, has its own unique ID or internet address. This setup allows all these different elements to identify each other, manage information, and share data back and forth, making it possible to collect and exchange details across a wide network. The information gathered from objects can be accessed through a range of devices, including mobile phones, computers, and tablets. The IoT is essentially the outcome of the convergence and advancement of three key components: the Internet, wireless technology, and microelectronic and mechanical systems. These networks have practically penetrated into all areas of people’s daily lives, the so-called smart lives, companies and society as a whole. These networks can be applied in industrial, agricultural, environmental, health, etc. sectors. Using the IoT in the healthcare sector can reduce costs and increase the accessibility and Quality of Service (QoS) that can be provided to patients [1–6].

In the health sector, IoT is used for monitoring the patients, helping the mobility impaired patients, and the rapid identification of patients. It is also applied in medication processes focusing on the home care services instead of expensive medical cares in hospitals. In these networks, first, the data are collected through a sensor on the patients’ body, and then, in order to process these data, they are sent to a user terminal such as a computer, smartphone, smart watch or even a special embedded device using some developed programs. The user terminal is linked via short-range communication protocols like Bluetooth. This gateway connects to the server service or the cloud data processing services [7–13].

Additionally, the health information system can keep patient data in electronic health records. This way, when a patient needs medical care, the doctor can easily access the patient’s medical history. Considering the use of IoT in patients’ health status monitoring in remote places, ensuring the data transmission quality is important in such a real-time environment because the data must be sent to the destination without any delay, especially in case of emergency, which necessitates a quick data delivery and fast routing. This way, the caregivers and doctors can become aware of the critical condition of the patients and provide timely services to them. On the other hand, since there exist some challenging limitations on network devices and equipment in terms of memory, processing power and battery, the routing algorithms should be proposed in view of these constraints. Optimal routing in the use of health care systems leads to a timely access to medical care, a decrease in healthcare expenses accompanied by an enhancement in the standard of healthcare provisions [14–17].

In this method which is introduced to monitor the diabetic patients and provide continuous care in treating them, sensors are placed on patients’ body to monitor their condition. These sensors send the patients’ data to medical centers. All the sensors are clustered, and each cluster is assigned a cluster head. If the medical center is in close proximity, the desired node will transmit the data directly, otherwise the data will be transmitted to the cluster head, which will subsequently relay the data to the medical center. To send the data, the Harris Hawks algorithm determines an optimal routing model. Correct routing is essential in the transmission of critical and timely data and helps save the patients’ lives.

To continue, different studies which have proposed algorithms for the IoT routing in the healthcare sector, are presented in the second part. Then, as shown in Table 1, these research works are compared with each other. In the third section, a background of the Grasshopper Optimization (GOA) and the Harris Hawks Optimization (HHO) algorithms is presented. In section 4, a method for optimal routing which can reduce the delay and energy loss, is introduced. In part 5, the suggested algorithm is simulated with MATLAB software and compared with other similar methods. And in section 6, the results and future works are stated.

**Table 1:** A comparison of the routing algorithms.

Delay	Complexity	Performance/ Efficiency	Network lifetime	Energy consumption	Key points	Year of publication	Algorithm
High	Low	Low	High	Low	Mamdani fuzzy model, Using parent-child relationship	2019	Chavva [12]
Low	High	High	High	Low	The presence of a queue Energy consumption and latency	2020	AFNDCAR [13]
High	High	High	High	Low	Whale optimization algorithm and fuzzy inference	2023	SIMOF [21]
High	Low	Low	Low	High	Route stability coefficient Route charges	2023	EPRS [22]
Low	Low	Low	High	Low	Prioritization and load balancing	2023	OE2-LB [23]
Low	High	High	Low	Low	Energy consumption and location	2018	Preeth [24]
High	High	Low	High	Low	Layering and clustering	2018	EEMA [25]
Low	High	High	High	Low	Two base stations and clustering	2019	E-HARP [26]
High	High	High	High	Low	Clustering, Pelican optimization algorithm, ranking	2023	Shyja [27]
Low	Low	Low	High	Low	Clustering and learning algoritm	2023	Jayabalan [28]
Low	Low	High	Low	High	Normal and emergency traffic	2023	MPR [29]
High	High	Low	High	Low	The modified gray wolf optimization algorithm	2023	DECR [30]

To discover an optimal routing model, the proposed method goes through the following steps

- Placing sensors inside the patients’ body
- Clustering the nodes using the GOA
- Assigning a cluster head to each cluster
- Cluster routing
- Sending the patients’ data to the medical centers

## 2. Review of literature

Fast and immediate response is a key concern in managing data flow within healthcare-related IoT networks. In order for the routing algorithms to have better efficiency, the parameters of delay, energy consumption and network lifespan should be taken into consideration in the application of the IoT in the healthcare industry [18]. To continue, the routing algorithms introduced in the healthcare sector are briefly reviewed and compared in Table 1.

To increase the network lifespan, [19] proposed a method through reducing energy consumption. In the introduced method, to frequently monitor the patients’ health, a routing technique was proposed using the Mamdani Fuzzy Algorithm. Moreover, based on the residual energy, a parent node was selected in a multi-step route so that the sensitive and critical data can be transmitted to the base station through these nodes. For network routing, [20] introduced the AFNDCAR (i.e., “adjusting forwarder nodes and duty cycle using packet aggregation routing”) algorithm which has a low latency. In the proposed method, a queue was formed for the next sending node, in which the probability of the success of the node in sending is calculated. The constituted queue was prioritized based on the energy consumption and delay. The node with the utmost priority was chosen to transmit the next step. In [21], an intelligent multi-objective fuzzy protocol, i.e., “SIMOF: swarm intelligence multi-objective fuzzy thermal-aware routing protocol for WBANs”, was introduced like a configurable network routing protocol within the intra-body networks. “SIMOF” consisted of two phases: (I) the Fuzzy Inference System (FIS) and (II) the Whale Optimization Algorithm (WOA) which automatically adjusts the rules. To select the suitable relay nodes, a multi-objective fuzzy inference system, which is based on the residual energy, distance, reliability, bandwidth, temperature, path losses and estimated energy consumption, was utilized in view of the IEEE 802.15.6 standard. To achieve the best performance in SIMOF, the Mamdani FIS rules were automatically adjusted using WOA. [22] proposed an algorithm to transmit the critical data, i.e., blood pressure, ECG and EEG, which are sensitive to delay. The “Enhanced Probabilistic Route Stability (EPRS)” protocol, as proposed, incorporates a novel cost function known as the Link Assessment Cost (LAC). This function plays a crucial role in evaluating the dependability of a route, thereby determining its suitability as a potential candidate for routing and fulfilling the QoS prerequisites. The Link Assessment Cost (LAC) relies on two crucial elements that determine the status of the link: (I) the Route Stability Factor (RSF) and (II) the likelihood of the expected cost of the link. E(p) Based on these factors, a score is assigned to a link that determines the status (the probability) of a link, i.e., being connected or disconnected. Therefore, despite the interruptions in the networks, the multi-dimensional EPRS selects the most stable and reliable routes, helps improve the route stability and throughput, and minimizes the end-to-end delay, route discovery and retransmission messages. To transmit the data to the next node, the algorithm suggested by [23], known as “opportunistic energy-efficient routing with load balancing (OE2-LB)”, determined the next node to be selected based on three conditions: (I) Sufficient capacity in the node buffer to receive the data, (II) The energy level of the node being no less than half of the initial energy, and (III) The delivery factor, as determined by the base station, being not lower than the specified threshold. This algorithm prioritized all his neighbors’ nodes. I t was shown that a node which meets all three conditions, has a high priority and can be selected as the next step node. In this method, to reduce the nodes’ delay and energy consumption, load balancing was used to select the next step node. In [24], to select the next step node, the network nodes were clustered based on the parameters of the source, the destination, and the number of steps to the destination. The assessment of these factors involved the consideration of resource placement models, including the event radius model and random source model. Although this method was efficient in reducing the energy consumption, it had a higher computational complexity. [25] introduced the “Energy-efficient multi-layered architecture (EEMA)” method. In this method, in each round, the network nodes were clustered, and a new cluster head was determined for each round. In the proposed architecture, the cluster head method was responsible for collecting the data from the lower layers. Then, in the higher layers, several clusters were combined to form superclusters, and the node in the central position of the cluster was responsible for the transmission of data to the base station. In the study conducted by the authors in [26], they utilized an “Energy Harvesting (EH)” mechanism along with a clustering algorithm that incorporated dual sinks. In this study, a total of fourteen sensor nodes and two sink nodes were employed within the human body as part of the experimental protocol. A clustering approach was used to avoid congestion at a sink node because the limited number of defined nodes could send the data to the specified sink node. The optimal sending node was chosen by the introduced protocol for every node, taking into consideration the optimal calculated value and the SNR of the link, as well as the required transmission power, distance, and the total available energy (including both the consumed and residual energies). Shyja et al. [27] proposed the “Optimal Link Quality and Energy Efficient Optimal Clustering-Multipath (LEOC-MP)” algorithm. The introduced method was implemented in three steps. First, to collect the data obtained from the body sensors, an optimal clustering technique was suggested using the improved Pelican Optimization (ICO) algorithm. Second, the rank of the node, energy efficiency, link quality, path loss, distance and delay were all computed using this method. Third, following the introduction of an automatic regression probabilistic neural network (AR-PNN) to optimize the design constraints and compute the cluster head (CH) for each cluster, the fish optimization (MPO) algorithm was employed to select multiple paths. This algorithm effectively identified the nearest, most efficient, and shortest node for transmitting pharmaceutical data. The method proposed by[28], utilized the Q-Learning to discover the best next step node to transfer the data. To receive the patients’ vital signs and send them to the medical centers, the introduced technique used a number of wearable sensor nodes. This method employed clustering to consume less energy.

In a research conducted by [29], a highly effective Multi-Path Routing Scheme (MPR) with a focus on QoS was put forward for the intra-body networks. In the context of MPR, the incoming traffic was categorized into two distinct groups: normal and emergency. The emergency traffic, which was assigned utmost priority and was routed through the most optimal route within the network, improved the reliability and throughput. The proposed model exhibited superiority over the advanced methodologies in various aspects, including energy efficiency, network throughput, package loss ratio, package delivery ratio, and end-to-end delay.

Next, as detailed in Table 1, we compare the methods mentioned earlier by looking at how well they perform, how complex their algorithms are, how much energy they use, how long they take to work, and how long the network can last using them. In [30], “the Distributed energy-efficient two-hop-based clustering and routing protocol (DECR)” method has been introduced. This method utilizes the modified gray wolf algorithm [31], incorporating the range of the node and the residual energy parameters, to effectively select the cluster head. Furthermore, a comprehensive analytical model has been suggested to ascertain an optimal number of clusters. This framework takes into consideration both the intra-cluster and inter-cluster transmission distances, in order to minimize the total transmission distances and the number of transmissions. For the purpose of routing, two modes are considered: intra-cluster and extra-cluster. In the intra-cluster mode, the nodes send their data to the nearest cluster head, while in the extra-cluster mode, the cluster head aggregates the total cluster data and transmits them to the base station. Table 1 presents a comprehensive analysis of various methodologies, focusing on key factors such as energy consumption in the entire network, the network lifespan, efficiency, complexity, and delay. The energy consumption parameter in the IoT of the body is a crucial metric owing to the network’s sensor-based nature and energy constraints. And the lifespan of the network is inversely related to the level of energy consumption. Moreover, the network performance metric associated with the throughput or transfer rate of data packages is the quantity of data packages that have been effectively transmitted. The algorithm’s complexity encompasses its time, memory, and computational aspects, as well as the final latency experienced when transmitting data packages to the intended recipients. Table 1 presents a comprehensive analysis of previous methodologies, highlighting the significance of these metrics within the network.

## 3. Background

In the proposed method, two optimization algorithms, i.e., Harris Hawks (HHO) and grasshopper (GOA), are used. Both the algorithms are introduced below.

### 3.1. An overview of the “grasshopper optimization algorithm”

The GOA which was first introduced in 2017, has been able to prove its efficiency and power in solving various problems [32]. Like the other optimization algorithms, this algorithm tries to find an optimal answer from among a number of solutions [33]. The grasshopper algorithm, renowned as one of the most potent optimization algorithms, can create a good balance between exploration and exploitation and provide an optimal approximation of the global optimum. The mathematical model of the proposed algorithm imitates the behavior of grasshoppers in nature to solve the optimization problem [34–36].

Clustering is employed to minimize energy consumption in the network and enhance control over the network nodes. In order to achieve this objective, the GOA is utilized. A tree structure is formed in the network to create clusters and determine cluster heads, enabling effective communication between the cluster head and the base station. The construction of the tree is facilitated by the implementation of the grasshopper algorithm within the base station. In fact, every individual grasshopper represents a tree and determines the arrangement of the cluster heads within the tree. A comprehensive description of the above process is presented below.

### 3.2. An overview of the “Harris Hawks algorithm”

The Harris Hawks algorithm which is a population-based, nature-inspired optimization paradigm, was introduced by Haidari et al in 2019 [37]. The main part of the brown hawk algorithm is derived from the collaborative nature and chasing tactics observed in Harris’s hawk, known as the “surprise attack”. In this intelligent approach, multiple hawks collaborate harmoniously to pursue a prey by ambushing it from various angles. In other words, the Harris’s hawk tries to surprise the prey by cooperating with several hawks and attacking from multiple direction [38]. Harris’s hawk exhibits various chase and pursuit styles, which are influenced by the dynamic scenarios and the escape tactics of its prey. In this approach, first, an initial population of hawks with different characteristics is generated. Based on the way this hawk surprises a prey while hunting, the position of the hawks is updated. To continue, different samples/examples are implemented on the obtained solutions in such a way that they can achieve better answers by consecutive iterations [39].

In the second stage, known as the routing stage, the data are transmitted by the cluster head to the medical centers. In this phase, the proposed approach uses the Harris Hawks algorithm to determine the next step node. The fitness rate of the neighbors within the radio range of the node is determined by evaluating their parameters through an analysis of the fitness function, alongside other potential candidates, and then, the most suitable node is selected as the next step. The subsequent explanation provides a comprehensive account of the way the Harris Hawks algorithm is utilized to calculate the compatibility/fitness of the nodes.

## 4. The proposed method

Using the Internet of Things (IoT) in healthcare allows for identifying many diseases from afar and even lets some treatments be done right at home. This way, it cuts down on the need to visit or be referred to hospitals often, making things more convenient and reducing the strain on healthcare services. Since Intelligentization of information registration systems has led to an increasing ease and accuracy in affairs, it is possible to predict, monitor and treat many diseases at a macro level at a faster speed. The introduced method which is proposed for an effective routing of nodes within the IoT network in the field of health, has two stages: (I) clustering and (II) efficient routing.

### 4.1. The clustering stage

In the proposed method, both the routing tree-based clustering and the GOA [40] are used for clustering in the body sensor network system. This approach uses a time-related variable mechanism that examines the behavior of nodes and a trusted grasshopper algorithm-based routing tree. The grasshopper algorithm presents itself as a viable option for clustering owing to its remarkable speed of convergence and the ability to swiftly update the location of mobile/moving nodes within the network.

**Fig 1:**
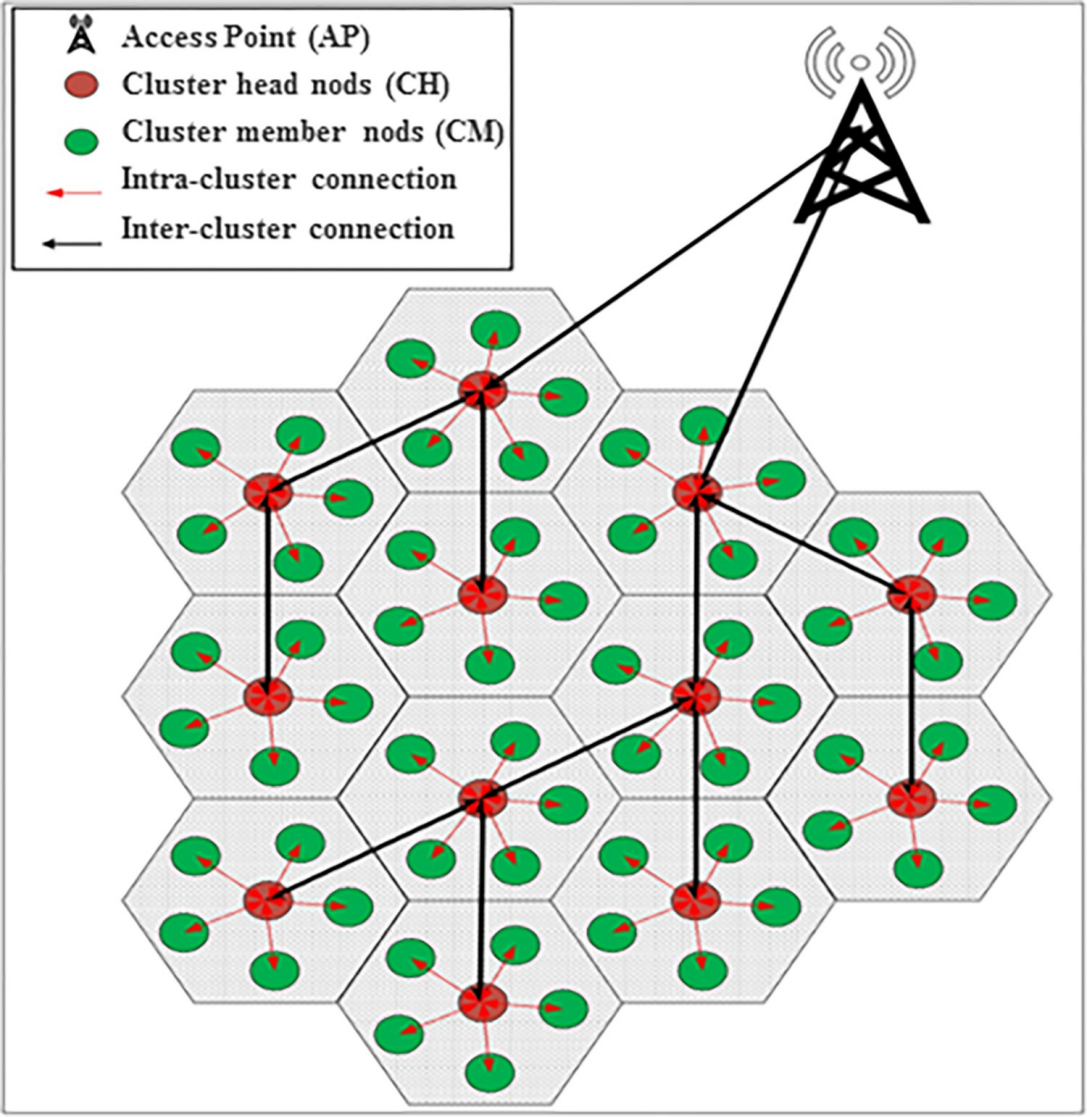
The routing tree-based clustering algorithm and the GOA [38].

The mechanism of this scheme is designed in such a way that it can divide the nodes into multiple clusters using the LEACH algorithm, and through rotation, select the cluster head from among the nodes in each cluster. To determine the nodes’ confidence coefficient, the behavior of nodes, when interacting with each other, is analyzed (Fig 1).

In [38], a network is organized into a tree structure to link the base station with the cluster heads. The base station employs the grasshopper optimization algorithm to create this tree. By periodically exchanging messages, it gathers detailed information about each cluster head, such as head node. its distance to the base station and its level of confidence and energy. Additionally, it puts all the cluster heads into a set. To form a population, the grasshoppers play the role of a tree and specify the arrangement of the cluster heads in the tree. In the population formation process, a cluster head is randomly selected from the set and its spatial coordinates are inserted into the corresponding element of the array. The first and second elements of the array are, respectively, considered the left and right children of each grasshopper. At each level of the tree, the left parent first identifies its left and right children based on the corresponding array. To evaluate the tree, a multi-objective fitness function is considered based on three parameters, i.e., the distance separating the cluster head from its primary node, the confidence level, and the cluster head’s energy. Then, based on this fitness function, the position of the grasshoppers is updated in each iteration. This update process aims to change the position of the cluster head in the routing tree and generate the most suitable tree. The base station is looking for a tree that can place a safer and more energetic cluster head at the higher level of the tree.

### 4.2. The efficient routing stage

**Fig 2:**
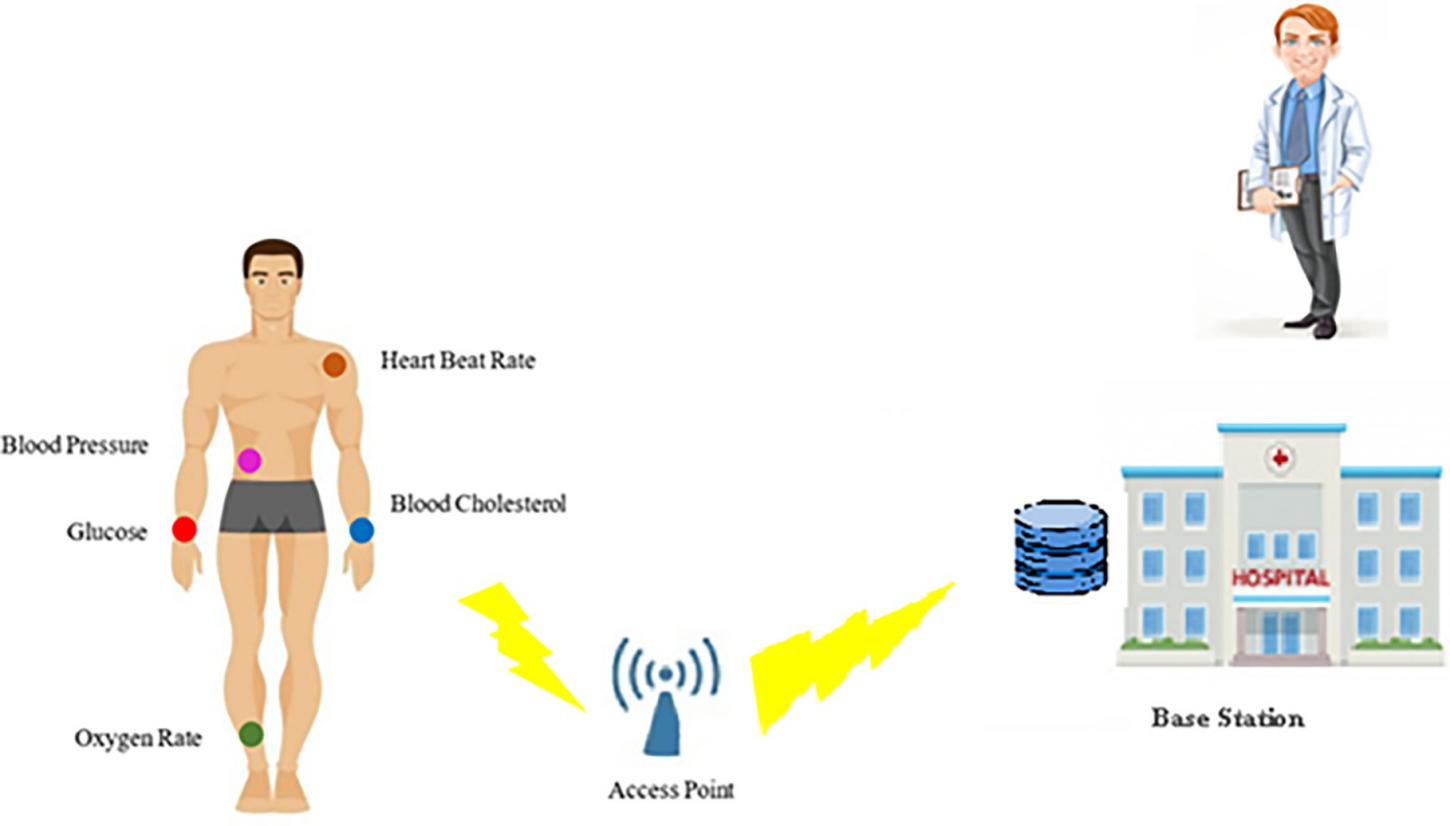
The placement of sensors in the body and the way the data is sent.

The concentration of glucose in the bloodstream of the patients with diabetes, whose pancreas is incapable of producing insulin, elevates. In the long term, high levels of glucose in the bloodstream have detrimental effects on the body, leading to the impairment and dysfunction of multiple organs and tissues. In the proposed method, to monitor and control the health of sick people, a number of sensors are embedded inside their body to measure various factors such as blood pressure, the level of glucose in the blood, heartbeat rate, the level of oxygen in the blood and the level of cholesterol in the bloodstream. Fig 2 shows the way these nodes are placed in the patients’ body.

The assumptions used in designing the proposed routing are as follows:

- In this approach, it is assumed that all the nodes possess an equal amount of initial energy.
- All the nodes have a unique ID.
- All the nodes have an equal number of sensors to measure the certain parameters.
- The coordinates of body sensors are fixed.
- Each node in the body will have mobility.
- Each node can be added to or removed from the network.

Each body contains five fixed sensors strategically placed on predefined locations on the person’s body. Once these sensors are affixed, they maintain mobility, moving in tandem with the body. This phenomenon is referred to as sensor mobility.

Nodes can be added to or removed from the network according to established protocols. Upon adding a new node to the network, it initiates the monitoring and transmission of sensors information through a designated sensor. Should the node cease data transmission, such as due to battery depletion or moving out of the receiver’s range, it transitions to an offline mode until returning to operational status, at which point it reverts to online mode. Nodes can also be manually removed from the network at the discretion of personnel.

The proposed method is an event-based one; when an event occurs, in-body sensors will report it to the medical centers and hospitals. For example, when a patient’s blood pressure is more than 18, his/her life may be endangered or even it may lead to his/her death; in such a critical situation, the patient’s medical condition will be reported to the medical center as soon as possible, and the hospital will contact his/her family to propose the necessary instructions to help improve his/her condition, i.e., recommending medicine with a specific dose. In this model, when a patient is alone without a companion, an ambulance will be sent to take him/her to a medical center.

In the IoT network, nodes are designed to respond to specific events and have a limited amount of energy available. This means it’s not practical to send data over long distances because it uses too much energy and isn’t cost-effective. Therefore, if the node is one step away from medical centers, it will send the data immediately in case an event occurs in the patient’s body, otherwise it will send it to the head of the cluster. The method of selecting the cluster head was investigated in the previous stage. To continue, routing by using the cluster head and sending data to medical centers are, respectively, examined in detail. In routing using Harris Hawks algorithm, an appropriate node is selected for the next step, which has several phases. Different stages of this algorithm to select the next step node are described below:

#### 4.2.1. Selecting the initial population

In the proposed approach, the Harris Hawks algorithm is employed to determine the optimal node for the next stage. To generate the initial population, all the neighbors of the source node are considered. The radio transmission range of the desired node is used to determine the neighbors of the node. Each neighboring node is considered as a solution.

#### 4.2.2. Calculating the fitness of Harris Hawks algorithm

In the introduced model, the fitness of each of the hawks is determined in the following manner:

$$f(X)=\alpha \times \frac{energy}{energy-total}+\beta \times \frac{packet-delivery}{packet-total}+\delta \times \frac{{Num}_{neighbors}}{Num-Total}$$ (1)

α+β+δ=1

To discover the best node of the next step, the value of f(x) is calculated for all the neighbors of the node.

Energy: the node’s residual energy in Joules

Energy-total: the node ’s initial energy level in Joules

Packet-delivery: the quantity of packages delivered by the node

Packet-total: the aggregate quantity of packages conveyed to the node

Numneighbors: the quantity of the node’s neighbors

Num_Total: the total quantity of nodes within the network

The node where the value of f(x) is higher, is selected as the next step node.

#### 4.2.3. Identifying the location of the prey

From among the population of the hawks, a solution that exhibits the utmost efficiency, is called the superior hawk, which possesses comprehensive knowledge regarding the hunting grounds. The location of the superior hawk is set as the hunting site.

#### 4.2.4. Updating the prey’s initial/primary energy source and jumping power

The initial energy and the jumping power of the prey are updated. In this step, these values will be used based on the relationships in calculating the prey’s energy expenditure and evaluating its success in escaping (diversifying the population of the hawks and generating new solutions for selecting server peers).


$$E_{0}={2r}_{5}-1$$ (2)

$$J=2(1-r_{5})$$ (3)

Where, r5 is a randomly generated number within the range of 0 and 1.

- ***Updating the Energy of the Prey***

The energy level of the prey is updated. The value of this parameter will have a great impact on the optimal selection of the next step node. In other words, based on this value, the Harris Hawks algorithm will select different strategies to reach a global optimum.


$$E=2E_{0}(1-\frac{t}{T})$$ (4)

If the parameter’s value is greater than or equal to 1 “(E≥1)”, the algorithm will enter the exploration phase, and new positions will be examined in the search space through the updating of the hawks’ location. This process offers new solutions for selecting the next step nodes. During this stage, the position of the brown hawks is updated.

If the parameter’s value is less than 1 “(E<1)”, the algorithm will be in the extraction or exploitation phase. In this phase, the four tactics of soft siege, hard siege, soft siege with fast dives and hard siege with fast dives are adopted by Harris Hawks. Using the mentioned strategies, the random solutions for the optimal selection of server peers are converged towards an optimal choice.


$$X(t+1)=\left\{ \begin{array}{l} \;X_{rand}(t)-r_{1}|X_{rand}(t)-2r_{2}X(t)|\;q\geq 0.5\\ (X_{rabbit}(t)-X_{m}(t))-r_{3}|LB)+r_{4}(UB-LB))\;q<0.5 \end{array}\right.$$ (5)

To approach and attack the prey, two parameters E and r are evaluated. The parameter r represents the prey’s chance of escape and the parameter E is the energy level of the prey. The variable r represents a randomly generated number within the interval of (0, 1).

If the values of r and E are both greater than or equal to 0.5, then the approach adopted will be that of a soft siege nature. Furthermore, the spatial vector will be updated by employing equations Eq (6).


$$X(t+1)=△X(t)-E|JX_{rabbit}(t)-X(t)|$$ (6)

If r is greater than or equal to 0.5 and E is less than 0.5, the strategy will be a hard siege type and the spatial vector will be updated using Eq (7).


$$X(t+1)=X_{rabbit}(t)-E|△X(t)|$$ (7)

If the value of r is less than 0.5 and the value of E is also less than 0.5, then the soft siege will be laid through quick dives and the spatial vector will be updated using Eq (8).


$$X(t+1)=\left\{ \begin{array}{l} Y\;if\;F(Y)<F(X(t))\\ Z\;if\;F(Z)<F(X(t)) \end{array}\right.$$ (8)

If the value of r is less than 0.5 and E is greater than or equal to 0.5, the hard siege will be laid through quick dives and the spatial vector of hawks will be updated using Eq (9).


$$X(t+1)=\left\{ \begin{array}{l} Y\;if\;F(Y)<F(X(t))\\ Z\;if\;F(Z)<F(X(t)) \end{array}\right.$$ (9)

#### Algorithm’s Termination Condition

**Fig 3:**
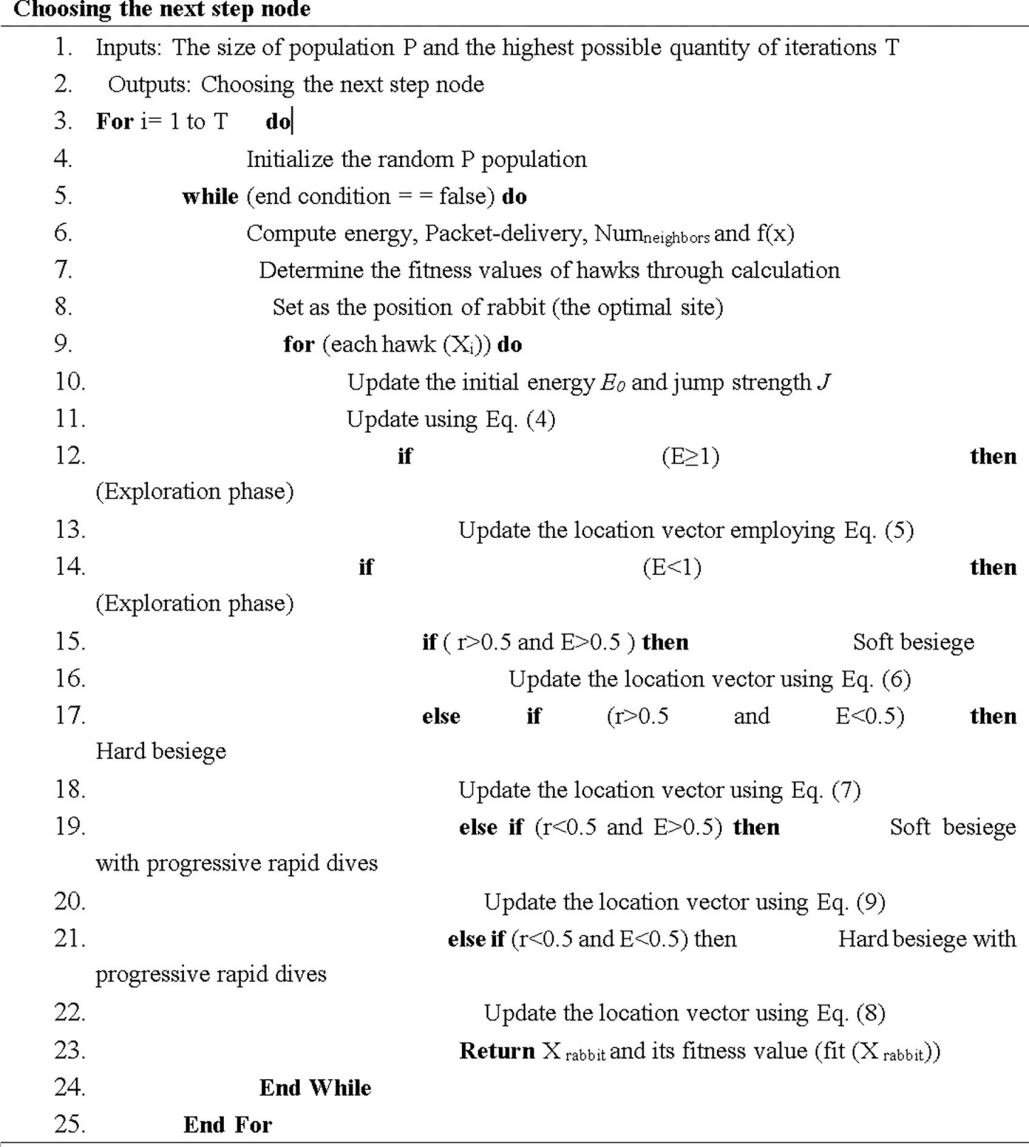
The pseudo code for determining the next step node.

After the Harris Hawks algorithm is executed for one round, it will be transferred to the next generation so that all the processes of the optimization algorithm can be executed again. This process will persist until the specified termination condition is satisfied. In the proposed method, the termination condition is the optimization algorithm’s execution times which will stop when the last round of the algorithm is executed. After the algorithm is terminated, the most efficient hawk will represent an optimal way to select an optimal node. As shown below, the pseudocode for choosing the next appropriate node has been presented in Fig 3.

First, the initial population, which is the neighbors of the node, is determined in line 4. As long as the end condition of the algorithm is not met, the algorithm will continue to run. Each node is considered as a hawk, and in the first round, its location is selected randomly. In line 7 which is the fitness function of each hawk, the node’s residual energy ratio, the ratio of the delivered packages, the number of the neighbors of the node, and the function f(x)’s value are calculated. The hawk that possesses the greatest level of fitness is commonly referred to as the superior hawk, which has detailed information about the hunting grounds. The position of the superior hawk can be set as a hunting position. In line 10, the initial energy and jumping ability of the prey are updated. In lines 12 and 14, the value of the energy parameter, which can be in the exploitation or exploration stage, is examined. Each hawk’s location is updated using Eq 5. Next, in lines 15 and 17, the prey’s energy and its chances of success in escaping are evaluated while it is under a hard or soft siege. Finally, all the hawks are fitted, the position of the prey is returned as an output, and the fittest node is selected.

#### The Proposed Method’s Flowchart

**Fig 4:**
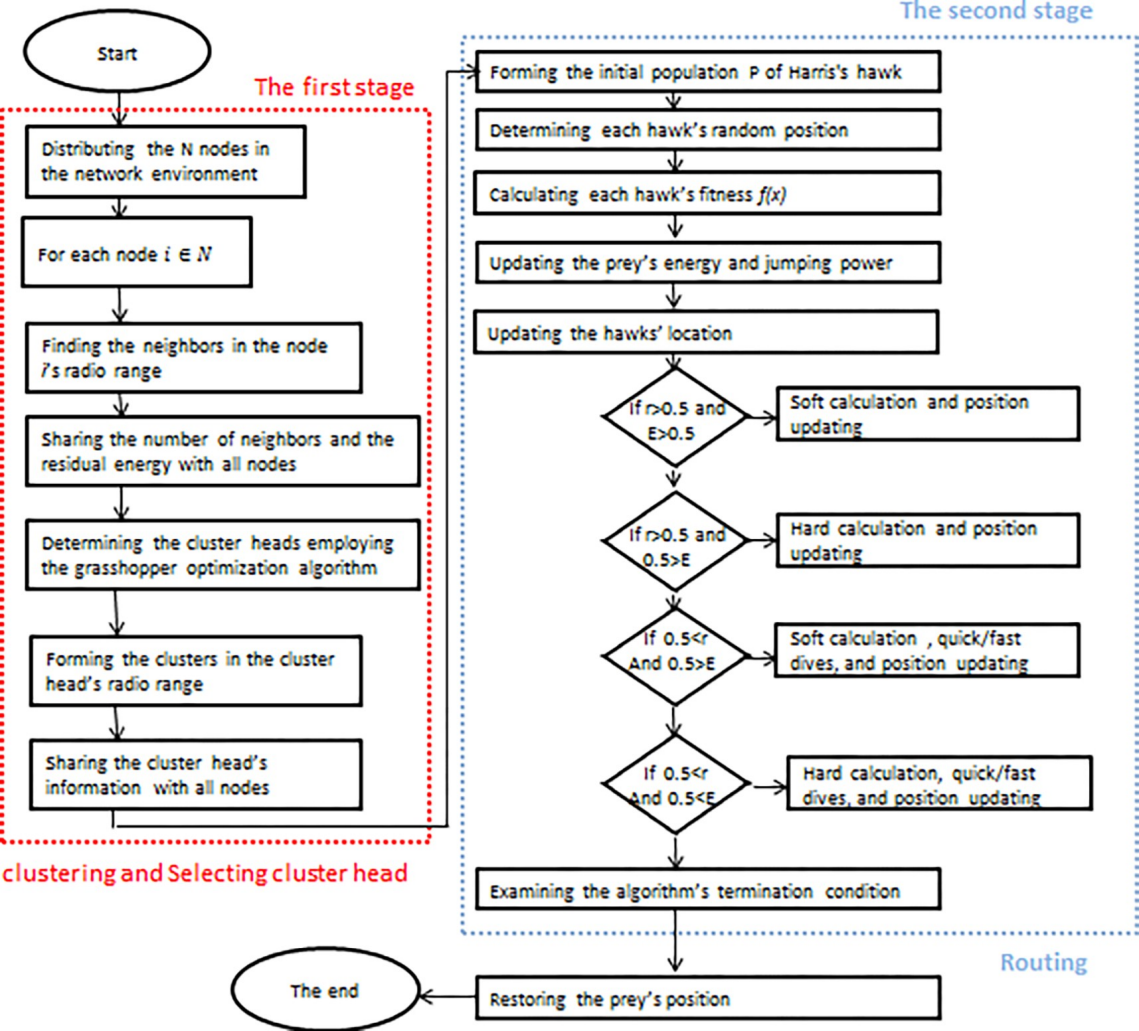
The proposed method’s flowchart.

The proposed method’s flowchart is presented below:

Fig 4 presents the flowchart of the introduced approach. Clustering is according to the level of the residual energy and the number of the neighbors. The radius of the nodes’ transmission range is used to estimate the number of the nodes’ neighbors. The GOA is applied to determine the cluster head. After the head of the cluster is determined and the clusters are formed, the second stage of routing is implemented using the Harris Hawks algorithm. Each neighboring node is considered as a hawk. The next step node is selected by the parameters of the node’s energy, the proportion of the quantity of packages that have been successfully transported and the number of the neighbors. The location of the hawk is randomly selected in the first round and its fitness is calculated. After the prey’s energy is updated, the location is reupdated according to the energy of the prey and its chance of escaping.

Once the cluster head is identified, the member nodes falling within its range are selected to form clusters. Subsequently, the details of each cluster head are broadcasted to all the nodes within the network. After the determination of the cluster head and the formation of the clusters, the second stage of routing is implemented using the Harris Hawks algorithm. Each neighboring node is considered as a hawk, and the initial population is formed. In the next step, the random position of each hawk is ascertained. The hawk’s fitness function is determined by evaluating the parameters of node energy, as well as the ratio of the number of delivered packages and the number of neighbors. The hawk’s location undergoes an update, alongside the prey’s energy level being updated as well. Once more, the location is updated in terms of energy and the chance of prey to escape; this process has 4 modes which will undergo a comprehensive examination. Here, the hawk concludes its endeavors to locate the next node to transmit the package, which, in fact, is the intended prey.

The algorithm will be executed until the end condition is met. At the end, the position of the fittest hawk is returned at the output. Below, the proposed approach is contrasted with alternative algorithms, and its results are shown in the form of diagrams.

## 5. The results of the simulation

The new algorithm designed for monitoring diabetic patients remotely can lessen the need for hospital visits, save time, and gather important health data to help determine the necessary insulin dosage. It supports the entire process of managing diabetes, from diagnosis and treatment to recovery and daily activity tracking, offering a comprehensive data management solution. This simulation assumes that the interaction between the network’s nodes involves several steps. The location and coordinates of the well and shipping rates are the program’s inputs. Energy consumption, package throughput, the end-to-end delay and other comparison parameters are the outputs of the program. The body sensor network model for remote controls in the workplace is presented in part 5–1. In part 5–2, the effectiveness of the suggested approach is assessed, and evaluation parameters, including the package delivery rate, the average end-to-end delay, the network throughput and energy consumption, are evaluated as the main factors to compare with other similar routing methods, i.e., QoS-Based Multi-Path Routing (QMPR) [29], QLearning [28] and EPRS [22], with the mentioned parameters.

### 5.1. The network model

**Fig 5:**
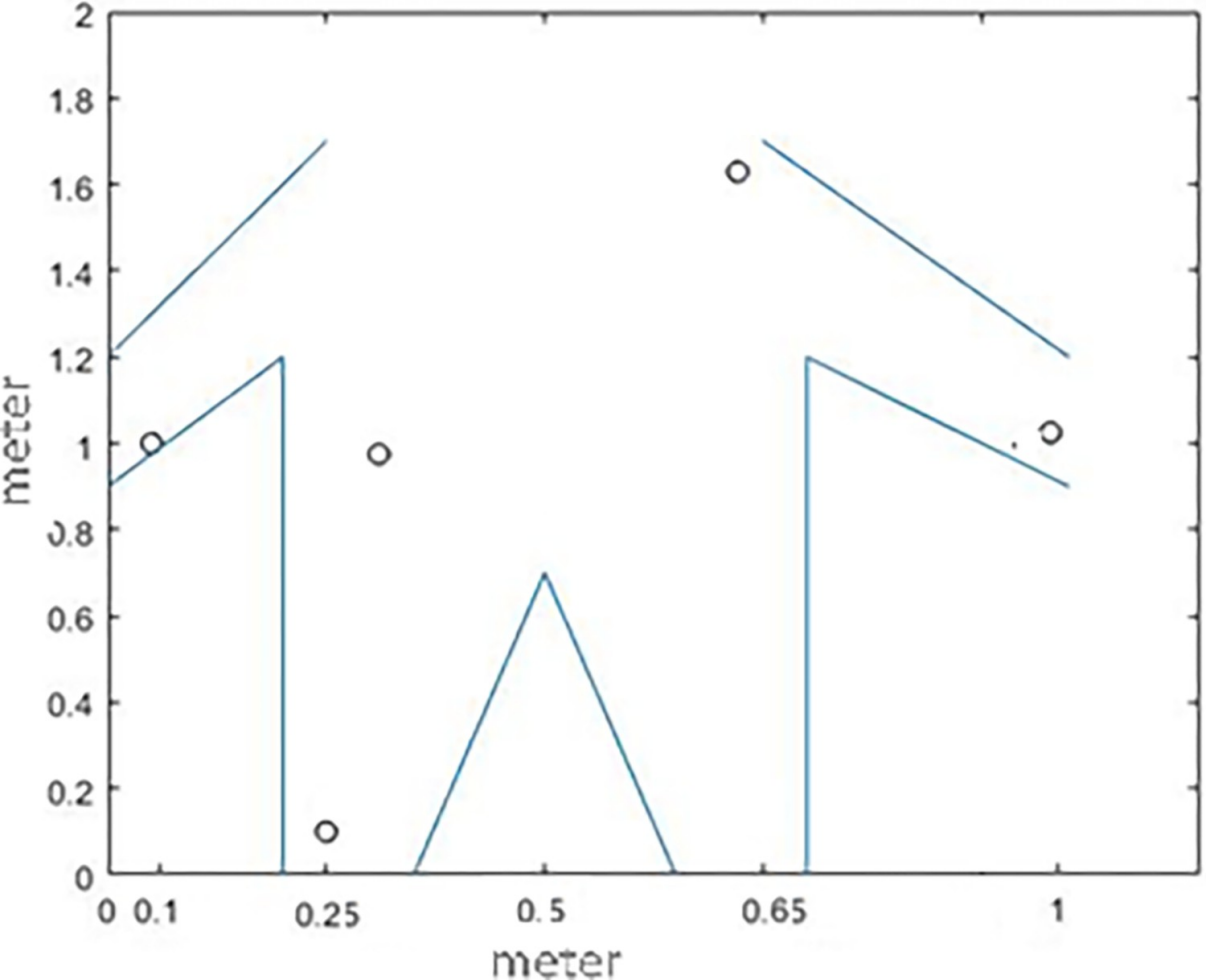
The fixed locations of sensors on patients’ bodies.

**Fig 6:**
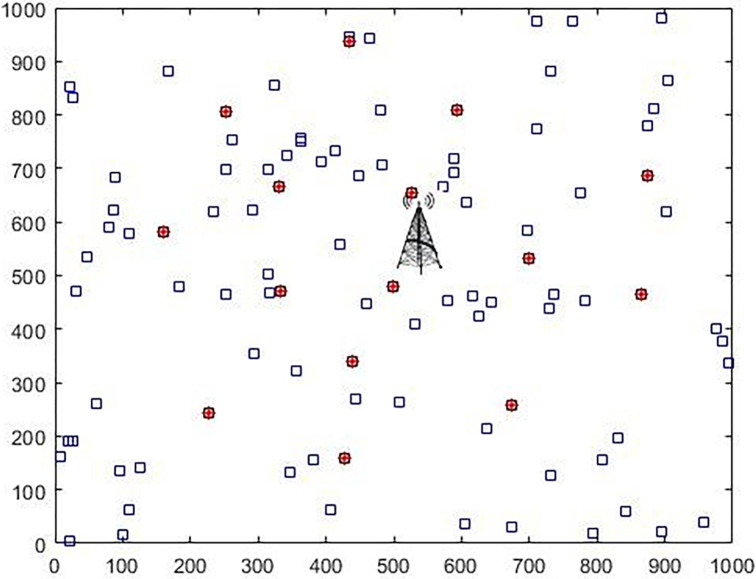
The network of body sensors, each node actually represents a person; the nodes send their data to the cluster heads. The task of the cluster head is to transfer information from the sensors to the base station.

An IoT network of the body is placed in a 1000x1000 square meter environment that is uniformly distributed and the nodes are randomly scattered in the IoT environment according to the specifications presented in Table 2. The simulation environment is MATLAB, which in this simulation, is situated at the center of the region, i.e., the 500 / 500 position of a data collection station. Fig 5, depicts a person with 5 sensors. These body sensors are positioned strategically to minimize potential damage to surrounding tissues, with fixed locations indicated. Measurements are provided in meters. The Fig 6 illustrates a network comprising red and blue dots, each representing a node. Each node corresponds to a person equipped with five sensors transmitting data Fig 5. The desired/given internet network is assumed to include the people with a limited energy source of body sensors. All these nodes have wireless communication. When individuals are in proximity to the access point, they can directly transmit data to the base station. Alternatively, if they are not within range, data is routed through selected cluster heads to the access point and then relayed to the base station. The clustering algorithm is utilized to select the cluster heads [38].

**Table 2:** The parameters used in the cluster simulation.

Values	Parameters
100 P	Number of people
500 N	Number of sensors
50 nJ/bit	Eelec
5 nJ/bit/signal	EDA
10 pJ/bit/m2	ƒs^*ε*^
0.0013 pJ/bit/m4	mp^*ε*^
4096 bits	Data package size
87 m	d0
0.5 J	The initial energy of nodes

The simulation is repeated for 8000 cycles, and the time period is 250 milliseconds. The consumed energy is calculated relying on the data presented in Table 2. The energy consumption framework and the nodes’ type exhibit similarities.

### 5.2. Evaluating the algorithm’s efficiency/ performance

MATLAB software is used in many industries and is extensively utilized in virtually any jobs and professions that necessitates data analysis. Given the significance of data and information in contemporary times, their role as crucial and strategic components in any setting/ environment cannot be overstated. Consequently, the ability to analyze data swiftly and effortlessly through MATLAB software holds immense value, as it facilitates effective mathematical and statistical computations. To effectively analyze data and address their issues, the majority of researchers are compelled to acquire proficiency in a programming language. This enables them to convert their problems into a computer language, leveraging the remarkable speed and precision of computers to solve these problems instead of relying solely on their own capabilities. Hence, acquiring proficiency in the language of communication with computers becomes imperative. Among these languages, programming with MATLAB software stands out.

To check how well the proposed method works, the software is set up on a computer system that runs on an OS 8.1 operating system. The system comprises an Intel (R) Core (TM) i7 processor operating at a frequency of 2.4 GHz, along with an internal memory capacity of 16 GB. The software environment utilized for this implementation is MATLAB 2018. As many as 100 individuals, who possess the ability to move freely, are randomly scattered in an area of 1000*1000 square meters. Every individual/patient possesses five sensors that are utilized to measure and monitor his/her blood pressure, body temperature, and blood sugar level. These sensors are strategically embedded in various coordinates of the person’s body, as depicted in Fig 5. The collected data are transmitted to the central facility where they are reviewed by the relevant medical professionals and specialists.

In these simulations, the effects of the proposed algorithm are investigated based on the metrics such as the average end-to-end delay, the throughput of data packages, energy consumption, and the network lifespan.

• The throughput of data packages: This metric is in fact the number of received packages ratio to the delay of data packages throughout the course of routing and transmission from the source to the destination. The network performance/efficiency is greatly influenced by it. The more the number of packages received at the destination and the more they pass through the routes with less delay, the higher the network throughput. Eq (10) shows the throughput of the network.


$$Average\;Throughput=\frac{sum\;of\;received\;packet}{delay}$$ (10)

• “The end-to-end delay”: The average period of time it takes for the data packages to be routed from the source to the destination, which is expressed in seconds. The average delay as the data packages’ retention time from the sender to the receiver is a measure of different networks. This time represents the cumulative duration of travel from the sender to reach the receiver by taking different steps from the relay nodes, which is calculated using Eq (11).


$$Average\;end-to-end\;delay=\frac{sum\;of\;time\;taken\;packet\;to\;recieve\;destination}{number\;of\;recieved\;packet}$$ (11)

• The energy consumption: This metric is an important parameter in determining the lifespan of the network of the body sensor internet The lifespan of the network increases and its efficiency improves as the energy consumption in the network nodes decreases. The energy consumption model in the IoT network, which in this simulation, is a type of sensor network, is directly related to the design of the media access control layer in these networks. However, if there exists a collective model that remains unaffected by the design parameters specified in the media access control layer, Eq (12) will be used to model the amount of energy consumption in these networks. Different assumptions about the characteristics of radio communication, such as energy loss during sending and receiving, can cause changes in the results of different protocols. In the proposed algorithm, the energy consumption model is employed based on the sender-receiver distance, similar to the utilization of the open space channel model (d2 energy loss) and multi-path (d4 energy loss). As a result, Eq (12) is employed to determine the quantity of energy expended in transmitting a package consisting of l bits over a distance of d.


$$E_{TX}(l,d)=E_{TX-elec}(l)+E_{TX-amp}(l,d)=\{\begin{array}{c} {lE}_{elec}+l_{\varepsilon fs}d^{2}\;d<d_{0}\\ {lE}_{elec}+l_{\varepsilon amp}d^{4}\;d\geq d_{0} \end{array}$$ (12)

Where, Eelec is the energy needed to initiate the electronic circuits, and $\varepsilon_{fs}$ and ε_amp_ are, respectively, the energies needed to enhance the signals for transmitting one bit in the open space and the multipath models. d is the sender and receiver nodes’ distance and d0 is the distance threshold value which is calculated using Eq (13).


$$d_{0}=\sqrt{\frac{\varepsilon_{fs}}{\varepsilon_{amp}}}$$ (13)

The energy consumed in the reception of a package consisting of l bit is calculated using Eq (14).


$$E_{RX}(l)=E_{RX-elec}(l)={lE}_{elec}$$ (14)

• The network lifespan: This metric is related to the amount of energy consumption; As the network’s energy consumption diminishes, the longevity of the network is enhanced. Therefore, the proposed protocols are all aimed at reducing energy consumption. The current investigation endeavors to enhance the longevity of the network through the selection of appropriate cluster heads and optimal routes. As the fewer nodes are involved in the data package sending route and the fewer steps are taken, the average energy consumption will be reduced.

#### 5.2.1. The network throughput

Throughput as one of the network evaluation parameters is so popular in the IoT networks. A maximum throughput indicates the high efficiency of the proposed method because when in the network, the data packages are not sent and the confirmation message is not received at the source, this node resends the mentioned data package and increases the network traffic and the total number of packages which are generated and sent in the network, which has been practically useless. Therefore, A greater throughput within the network signifies the effective utilization of the network’s resources, encompassing the bandwidth as well as the limited energy resources within the nodes. Determining appropriate cluster headers and shipping routes in the network has resulted in a high efficiency of the proposed approach (as shown in diagram 7). This approach avoids generating a duplicate data package and decreases the network traffic and the package loss.

**Fig 7:**
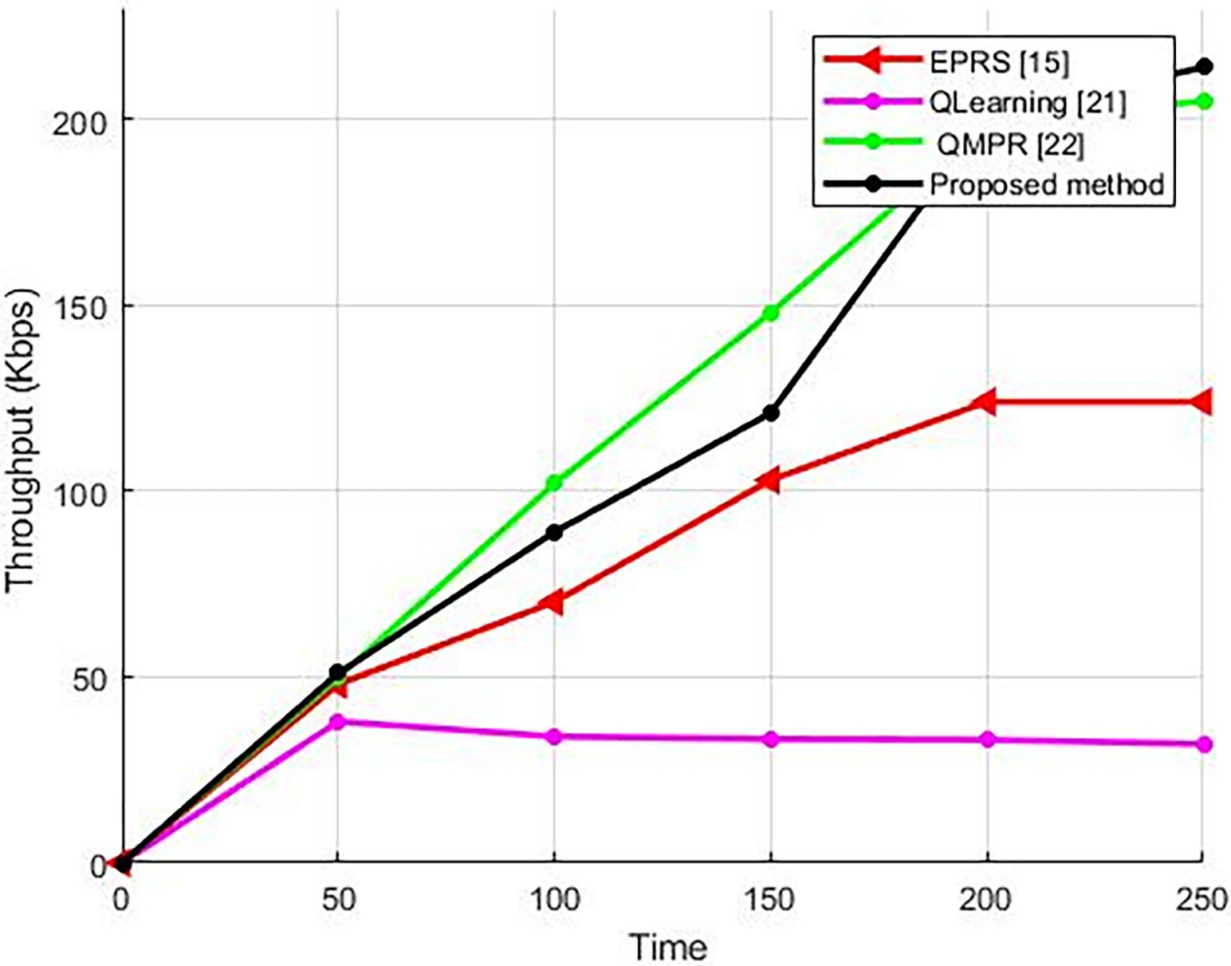
The body sensor network’s average throughput in the proposed method compared to different algorithms.

Fig 7 illustrates the network throughput in the suggested technique as well as alternative methodologies, which is directly related to the package delivery rate. The methods in which the delivery rate is higher compared to the other approaches, have a higher throughput; These methods do not encounter the failed transmission of data packages by the nodes, and the data packages are sent from safe routes to ensure they are delivered to their destination.

#### 5.2.2. The mean end-to-end latency

**Fig 8:**
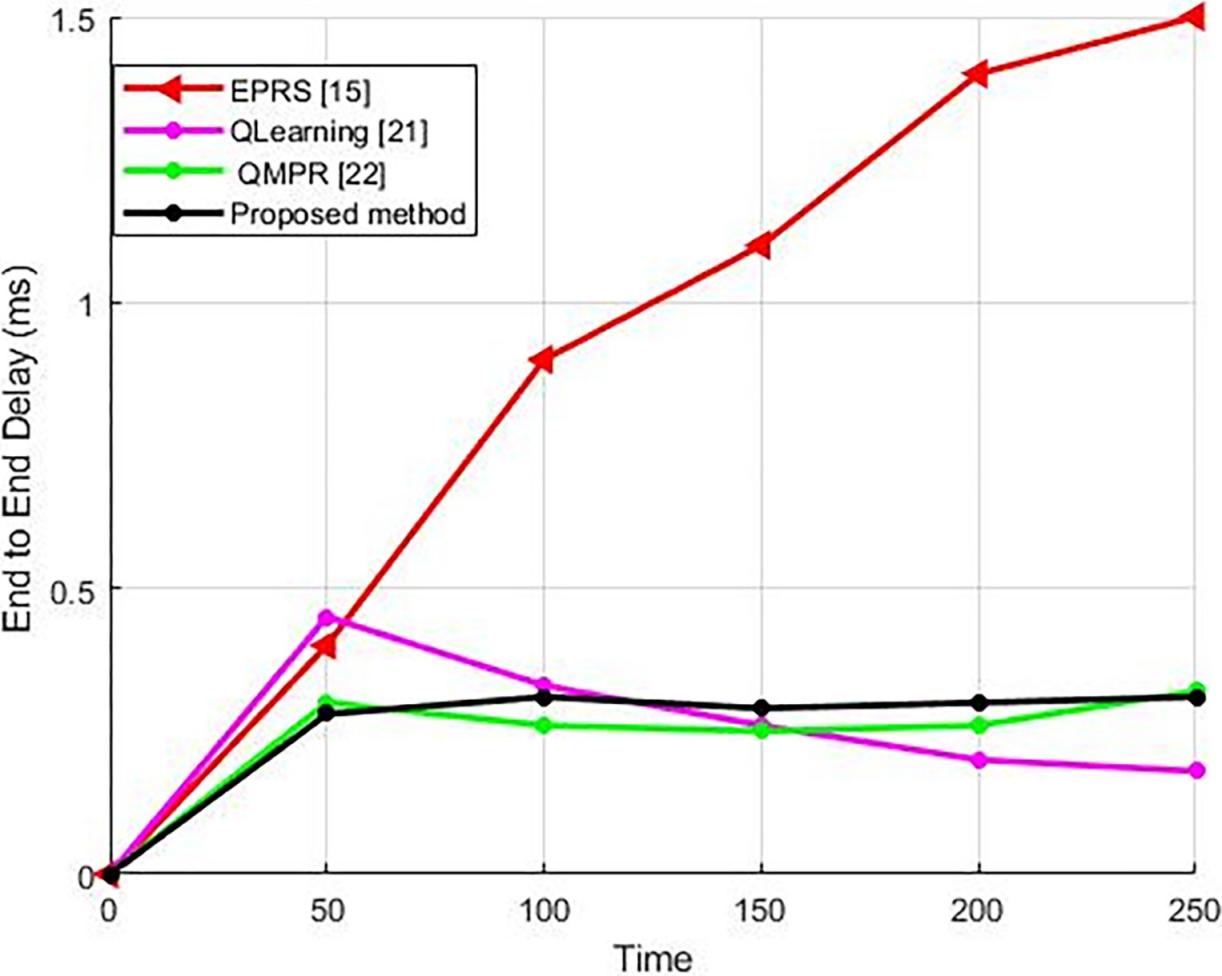
The algorithms’ average end-to-end latency.

The duration it takes for a package to travel from its source to its intended destination is referred to as the end-to-end delay. The average end-to-end delay corresponds to the average time it takes for packages to travel from the source to the destination within the network. As shown in Fig 8, compared to other approaches, in the proposed method, the average delay in the network is lower because the efficiency and throughput of the introduced method is higher than the other techniques; and due to the reduced traffic within the network, the average duration for delivering packages in the network as well as the waiting time for data packages at network nodes have both experienced a decrease.

A rise in the network traffic leads to an increase in the end-to-end latency in the network nodes, and as a result, as long as the package is alive, it will remain in the intermediate node’s buffer until it is sent, which causes the package to be discarded at another node because the package survival time is over, which increases the average end-to-end latencyin the network nodes. Therefore, applying the right approach to routing in the network will reduce the average end-to-end latency. In fact, reducing the time taken to deliver data packages within the network is an objective that is highly sought after. The primary objective of the proposed approach is to minimize the average end-to-end latency within the network, a crucial parameter. This optimization is particularly significant in time-sensitive scenarios such as emergency situations and real-time applications, where prompt delivery of packets to their intended destination is of utmost importance.

Over time, the proposed method exhibits a higher average end-to-end delay in comparison to the QMPR [29] and QLearning [28] algorithms because in contrast to the above algorithms, the throughput of the introduced method experiences a reduction. However, compared with the proposed method, these algorithms consume more energy. As stated before, one of the important goals of the proposed method is to reduce energy consumption due to the IoT network’s inherent energy limitations. If energy consumption is not given priority by the proposed method, it will have the potential to achieve the highest throughput and the lowest average end-to-end delay. The proposed method applies the energy parameter through the merit function of the HHO algorithm.

#### 5.2.3. The comparison of energy consumption between the proposed algorithm and other methods

**Fig 9:**
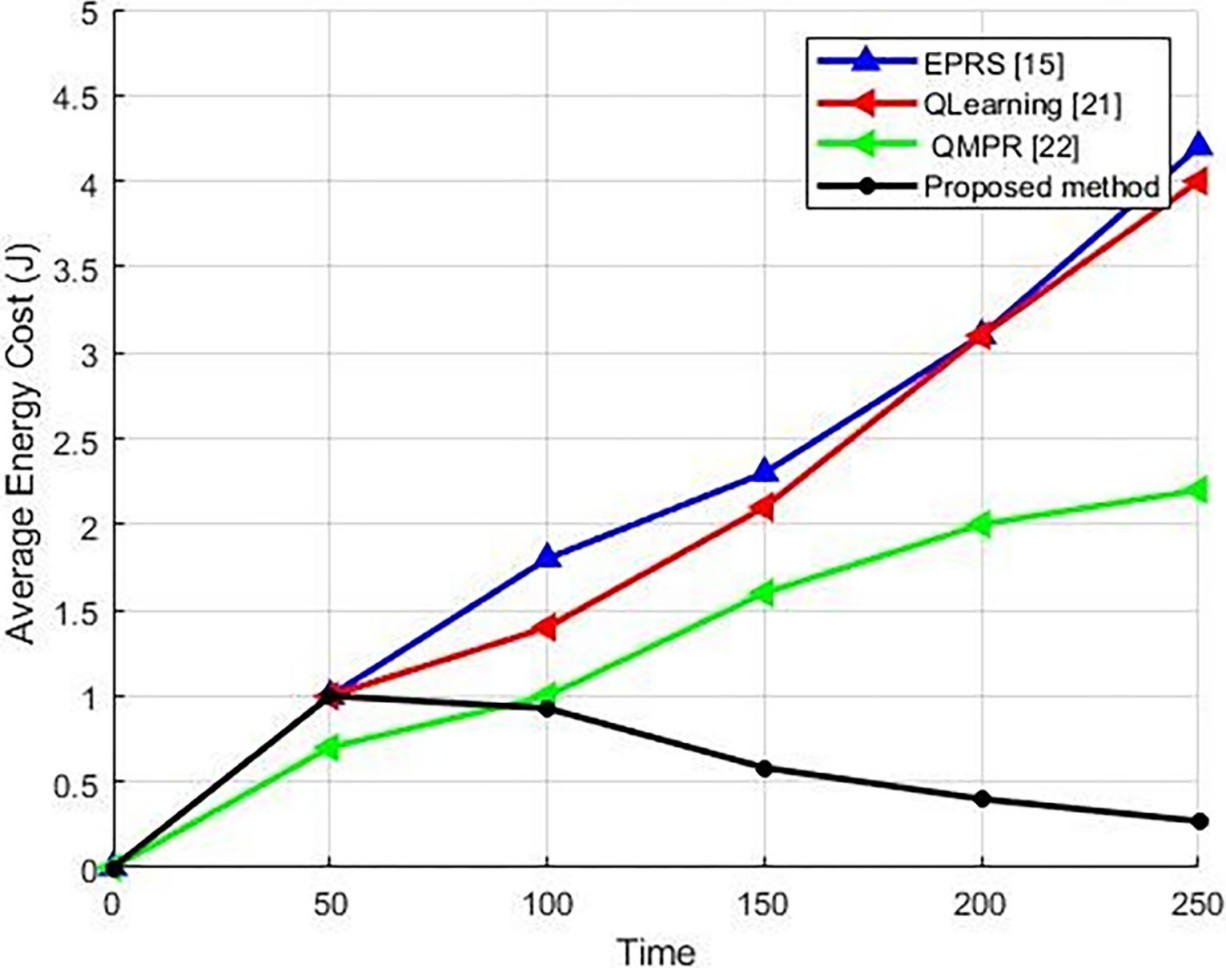
A comparison of energy consumption in different methods.

Energy is an inseparable and important parameter in the IoT networks. Energy efficiency is one of the main design goals in the development of IoT protocols in the body sensors. While energy limitations are the main issue in the medium access control (MAC) or network layer, the number of transmitted packages is also involved in the total energy consumption. Since energy efficiency is an important metric for any IoT network protocols,. the efficacy of the suggested methodology is evaluated with regards to energy usage. As shown in Fig 9, the energy consumption has an upward trend until 50 milliseconds because as long as the system does not find the right path, the data package delivery rate will be lower and more packages will be lost, which will result in resending the same package and, once more, an energy loss.

As shown in the package delivery rate graph, the number of the lost data packages is reduced. With each package drop, the energy is lost twice. In a failed transmission, not only is energy wasted, but also for retransmission, an additional battery power may be required at several times. In order to enhance energy efficiency and prolong the lifespan of the network, the proposed approach lowers the number of package shipments and tries to determine the optimal path and appropriate cluster heads.

Fig 9 depicts the energy consumption diagram of the proposed method in comparison to the alternative approaches. Over time, the proposed method stabilizes the route, the optimal clusters, and the cluster heads. Consequently, data packages are transmitted through this route, regardless of any potential delays in the desired route. Therefore, as time progresses, the graph no longer requires the essential energy to ascertain the cluster head, the cluster, and the optimal route, resulting in a reduction in energy consumption. However, in alternative approaches, different mechanisms are employed for the purpose of routing, resulting in significant energy consumption.

**Fig 10:**
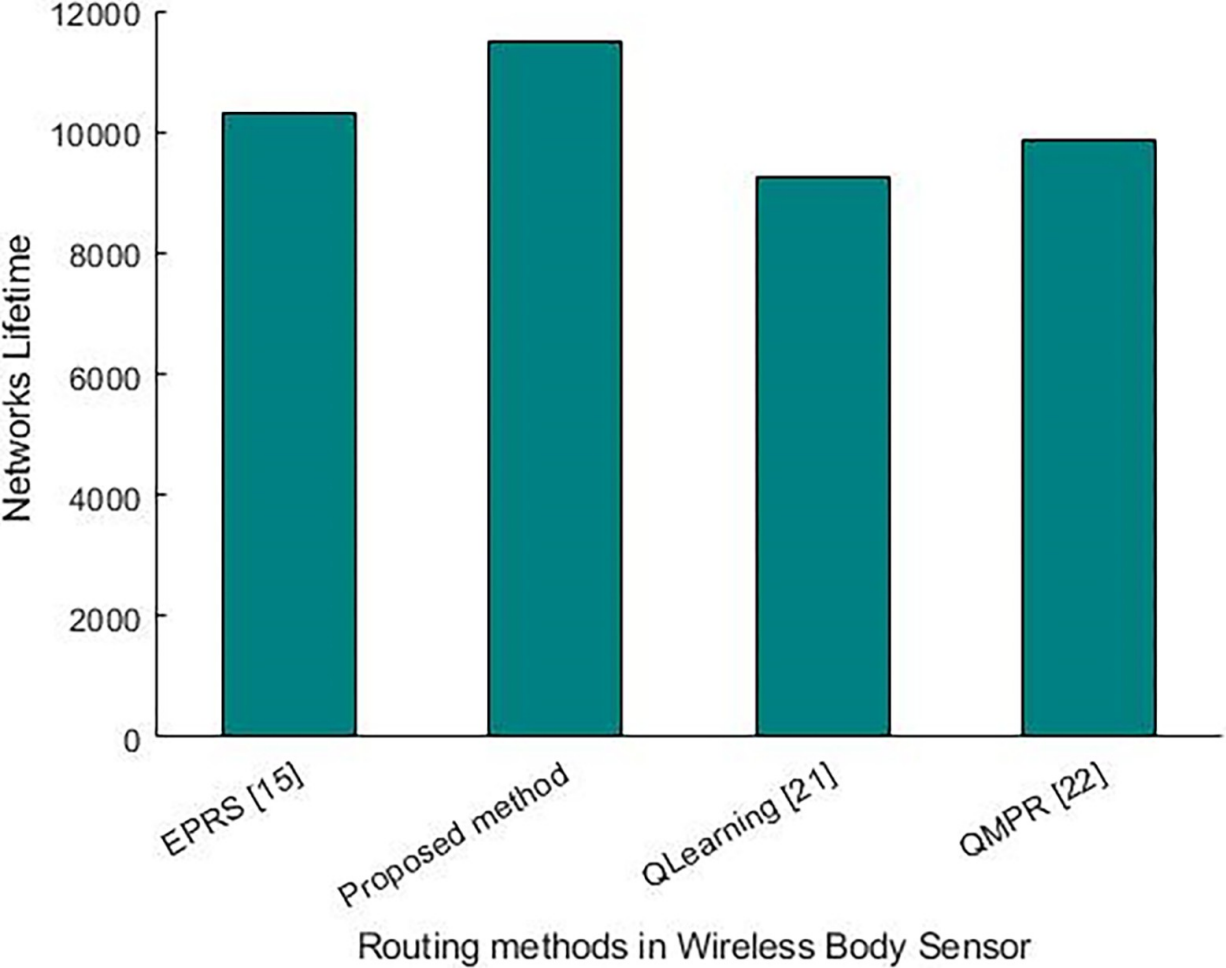
A comparison of the introduced method with other existing methods in terms of the network lifespan.

The network lifespan is another metric which is inversely related to energy consumption. Fig 10 which shows the lifespan of the network, illustrates an improvement in routing, an appropriate determination of cluster heads and an optimal clustering.

The lifespan of the network is assumed based on round in networks; It is postulated that each round, encompassing network operations such as data transmission, reception, and routing, lasts for 100 milliseconds. Therefore, each unit in the diagram is equivalent to 100 units of time. In previous studies and related literature, it is crucial to consider the round unit.

## 6. Conclusion

The IoT networks which are one of the emerging technologies, have significant applications in various fields, including health, treatment and medical services. These networks which can manage the diseases, have a significant impact on the recovery process of patients through focusing on the follow-up processes and an early diagnosis and treatment of the diseases. Also, they can reduce the treatment costs. These networks can record the detailed information about the pressure of the blood, the level of glucose in the bloodstream, heart rate, the blood oxygen level, and the blood cholesterol level for long periods of time. Considering the increasing incidence of diabetes and prevalence of other diseases, this study proposes the IoT system that is effective in controlling the diseases. In the proposed method, the GOA is employed to cluster the network nodes. In the process of clustering, the initial step involves the determination of the cluster head, and then, the nodes within the radio range of the cluster head are placed in a cluster. In the second step, employing the Harris Hawks algorithm, the next step node assesses the fitness value of each node for the next step based on the node’s remaining energy ratio, the ratio of delivered packages, and the number of the node’s neighboring nodes. The proposed method is assessed by comparing its efficiency, energy consumption, and delay with other algorithms.

Given that the proposed method has been closely studied in actual medical routing applications, it shows improvements in reducing delays between points and lowering energy use. The proposed algorithm exhibits a remarkably low point-to-point delay, particularly during subsequent iterations or higher rounds, resulting in a notable improvement of 33%. While the delay parameter in the proposed method exhibits a gradual increase in slope in subsequent iterations or higher rounds, in alternative approaches, the delay escalates rapidly. Energy consumption, a crucial parameter in medical applications, demonstrates a notable enhancement of approximately 60% in subsequent iterations or higher rounds, without experiencing an upward and sudden increase as the number of executions or rounds increases. The method exhibits a gradual/gentle increase in the slope of its graph, which is considered a favorable aspect of the proposed approach.
